# Psychopathology and Stem Cell Mobilization in Ultra-High Risk of Psychosis and First-Episode Psychosis Patients

**DOI:** 10.3390/ijerph19106001

**Published:** 2022-05-15

**Authors:** Katarzyna Waszczuk, Jolanta Kucharska-Mazur, Ernest Tyburski, Katarzyna Rek-Owodziń, Piotr Plichta, Krzysztof Rudkowski, Piotr Podwalski, Tomasz Grąźlewski, Monika Mak, Błażej Misiak, Anna Michalczyk, Maciej Tarnowski, Katarzyna Sielatycka, Angelika Szczęśniak, Karolina Łuczkowska, Barbara Dołęgowska, Marta Budkowska, Mariusz Z. Ratajczak, Jerzy Samochowiec

**Affiliations:** 1 Department of Psychiatry, Pomeranian Medical University in Szczecin, Broniewskiego 26, 71-460 Szczecin, Poland; jola_kucharska@tlen.pl (J.K.-M.); krudkowski@gmail.com (K.R.); piotr.podwalski@gmail.com (P.P.); tgrazlewski@gmail.com (T.G.); annakarolina6@wp.pl (A.M.); samoj@pum.edu.pl (J.S.); 2 Department of Health Psychology, Pomeranian Medical University in Szczecin, Broniewskiego 26, 71-460 Szczecin, Poland; ernest.tyburski@gmail.com (E.T.); katarzynarek90@gmail.com (K.R.-O.); piotrpp119@gmail.com (P.P.); monika.mak@gmail.com (M.M.); 3 Department of Psychiatry, Division of Consultation Psychiatry and Neuroscience, Wroclaw Medical University, 50-367 Wroclaw, Poland; mblazej@interia.eu; 4 Department of Physiology, Pomeranian University of Medicine, Powstańców Wielkopolskich 72, 70-111 Szczecin, Poland; maciej.tarnowski@pum.edu.pl; 5 Institute of Biology, Faculty of Exact and Natural Sciences, University of Szczecin, Felczaka 3c, 71-415 Szczecin, Poland; katarzyna.sielatycka@usz.edu.pl; 6 Department of Laboratory Medicine, Pomeranian Medical University in Szczecin, Powstańców Wielkopolskich 72, 70-111 Szczecin, Poland; angelika.szczesniak.92@gmail.com (A.S.); barbara.dolegowska@pum.edu.pl (B.D.); 7 Department of General Pathology, Pomeranian Medical University in Szczecin, Powstańców Wielkopolskich 72, 70-111 Szczecin, Poland; karolina.luczkowska@pum.edu.pl; 8 Department of Medical Analytics, Pomeranian Medical University in Szczecin, Powstańców Wielkopolskich 72, 70-111 Szczecin, Poland; marta.budkowska@pum.edu.pl; 9 Stem Cell Institute at James Graham Brown Cancer Center, University of Louisville, Louisville, KY 40292, USA; mariusz.ratajczak@louisville.edu

**Keywords:** stem cells, HSCs, VSELs, embryonic stem cells, high-risk psychosis, UHR, first-episode psychosis, schizophrenia

## Abstract

Although regenerative and inflammatory processes are involved in the etiopathogenesis of many psychiatric disorders, their roles are poorly understood. We investigate the potential role of stem cells (SC) and factors influencing the trafficking thereof, such as complement cascade (CC) components, phospholipid substrates, and chemokines, in the etiology of schizophrenia. We measured sphingosine-1-phosphate (S1P), stromal-derived factor 1 (SDF-1), and CC cleavage fragments (C3a, C5a, and C5b-C9; also known as the membrane attack complex) in the peripheral blood of 49 unrelated patients: 9 patients with ultra-high risk of psychosis (UHR), 22 patients with first-episode psychosis (FEP), and 18 healthy controls (HC). When compared with the HC group, the UHR and FEP groups had higher levels of C3a. We found no significant differences in hematopoietic SC, very small embryonic-like stem cell (VSEL), C5a, S1P, or SDF-1 levels in the UHR and FEP groups. However, among FEP patients, there was a significant positive correlation between VSELs (CD133+) and negative symptoms. These preliminary findings support the role of the immune system and regenerative processes in the etiology of schizophrenia. To establish the relevance of SC and other factors affecting the trafficking thereof as potential biomarkers of schizophrenia, more studies on larger groups of individuals from across the disease spectrum are needed.

## 1. Introduction

Stem cells (SCs) are a unique kind of non-specialized cells that can self-renew and differentiate into other types of cells. Depending on their ability to differentiate, SCs can be divided into (1) totipotent, which can transform into any other kind of cell, including extraembryonic tissues such as the placenta; (2) pluripotent, which can develop into all three germ layers but not the tissues of the placenta; (3) multipotent, with the ability to differentiate into one of the germ layers (ectodermal, endodermal, or mesodermal); or (4) unipotent, also known as tissue-targeted cells, which give rise to only one cell lineage [1]. Based on evolutionary stages, SCs can be classified as embryonic, fetal, infant, perinatal, or adult cells [2]. Ratajczak et al. isolated another homogenous population of SCs that exhibits characteristics of embryonic SCs and the capability to differentiate into all three germ layers. They are known as very small embryonic-like stem cells (VSELs) [3,4,5]. 

For decades, SCs have generated increasing interest due to their potential use in exploring the roots of different diseases, toxicity tests for new drugs, and regenerative medicine [6]. Currently, bone marrow hematopoietic stem cell (BM-HSC) transplants are used to treat, inter alia, multiple myeloma, various kinds of leukemias, germ cell tumors, and aplastic anemias [7]. BM-HSCs, bone marrow mesenchymal stem cells (BM-MSCs), umbilical cord MSCs, adipose stem cells (ASCs), mesenchymal precursor cells, and fetal stem cells are used to treat type 1 and 2 diabetes mellitus [8]. BM-MSCs, ASCs, synovial tissue-derived mesenchymal stem cells, peripheral-blood-derived progenitor cells, and bone marrow concentrate are used in the treatment of different kinds of musculoskeletal injuries (e.g., osteoarthritis–cartilage defects, femoral head osteonecrosis, and osteogenesis imperfecta) [9]. There are also promising results for neurological and neurodegenerative disorders, such as brain ischemia, multiple sclerosis, and possibly even Huntington’s and Parkinson’s diseases [10]. Moreover, new potential therapies are being investigated, such as the use of VSELs in myocardial infarction [11,12].

Some organs contain both fast-cycling SC populations, responsible for fast cell turnover, and slow-cycling SC populations, necessary to maintain tissue after injury; organs with slow cell turnover, such as the brain or liver, contain only slow-cycling SCs [13]. Although in small numbers, there are various tissues from which SCs can be extracted, such as bone marrow, adipose tissue, the retina, cerebral cortex, small intestine, etc. [14,15]. VSELs can be found in bone marrow, peripheral blood, and umbilical cord blood, as well as various adult organs, such as the brain, kidneys, and pancreas [16]. To date, increased numbers of VSELs have been confirmed after stroke [17] and myocardial infarction [18].

There are still few reports concerning the role and usage of SCs in psychiatry, even though mental disorders are a growing public health problem with a rising cost to the world economy [19]. One such illness is schizophrenia—a debilitating chronic disease, characterized by disturbances in perception, thinking, and behavior. Despite its relatively low prevalence, it is a significant cause of disability among people of working age [20]. According to retrospective studies, even up to 73% of patients may experience concerning symptoms a few years prior to their first hospitalization—typically depressive or negative symptoms [21]. Early detection and intervention are crucial when dealing with a prodromal period of, on average, several years before the first admission to the hospital.

Abnormal hippocampal neurogenesis has been proposed to play a role in the etiology of schizophrenia [22,23], supporting the neurodevelopmental model of the disease [24,25]. Given that neurogenesis is impaired in adults, SCs could potentially be used as a biomarker of a developing mental condition such as schizophrenia. Initial research has shown that the concentration of VSELs is increased in first-episode psychosis (FEP) patients [26]. Higher levels of VSELs, MSCs, and endothelial progenitor cells have also been shown in patients with bipolar disorder not taking lithium [27]. In panic disorder, the levels of HSCs and VSELs have been found to be significantly lower than in control groups [28]. All of the aforementioned research provides insight into the regeneration processes occurring in mental disorders and could be helpful in the diagnosis and differentiation of various mental disturbances.

Moreover, as SC trafficking is controlled by various factors—including phospholipid substrates such as sphingosine-1-phosphate (S1P), the growth factor chemokine stromal-derived factor 1 (SDF-1) and complement cascade (CC) cleavage fragments (e.g., C3a, C5a or C5b-C9; also known as the membrane attack complex) [29]—it is also important to analyze the role of these factors in the etiopathogenesis of schizophrenia. To our knowledge, this is the first study to assess both SCs and factors affecting the mobilization thereof among individuals with an ultra-high risk of psychosis (UHR) and FEP patients.

The aims of this study were (1) to check whether changes in the concentration of HSCs, VSELs, and factors affecting the mobilization thereof in peripheral blood are present at the initial stages of the psychotic spectrum (UHR individuals and FEP patients), and (2) to assess if there is any relationship of severity of symptoms with HSCs, VSELs, and factors influencing the mobilization thereof. Based on the existing literature, we hypothesized that (1) the number of HSCs, VSELs, and factors affecting the mobilization thereof in the peripheral blood will differ between UHR, FEP, and the control group, and (2) the severity of symptoms will correlate with the concentration of SCs and factors affecting the mobilization thereof.

## 2. Materials and Methods

### 2.1. Participants

We recruited 49 unrelated patients—9 UHR individuals, 22 FEP patients—from the Clinic of Psychiatry of the Pomeranian Medical University in Szczecin, and 18 healthy controls (HC) who volunteered to participate in the study in response to advertisements disseminated online and amongst the students who are taught at the clinic. Recruitment occurred in the inpatient clinic, outpatient clinic, and daycare unit of the university. Diagnosis of UHR was based on the Structured Interview for Prodromal Syndromes (SIPS) [30,31], while FEP patients had clinical symptoms of non-affective psychosis (F20, F22, F23), diagnosed in accordance with the International Statistical Classification of Diseases and Related Health Problems [32] and confirmed with the use of the Mini-International Neuropsychiatric Interview [33]. The following were inclusion criteria: 18–40 years of age, comprehension of the test procedure, and a stable physical state. Exclusion criteria included comorbid mental disorders, an evident coincidence of symptoms with psychoactive substances, severe somatic diseases, and active inflammatory disorders. Due to the small sample size, one FEP patient aged 41 was included. A total of 18 healthy participants with no mental or neurological disorders, confirmed by evaluation by trained psychiatrists and a structured self-report questionnaire, were recruited as a control group. They were matched in terms of sex and years of education. The exclusion criteria did not differ from the patient group. The study protocol was approved by the Ethics Committee of the Pomeranian Medical University in Szczecin. 

### 2.2. Clinical Assessment

The research procedure included examination by trained psychiatrists and laboratory tests. A structured interview was used to collect demographic information, history of symptom evolution and potential treatments, and family history from patients. The Positive and Negative Syndrome Scale (PANSS) was used to assess the FEP patients’ psychopathological functioning [34,35]. All items were scored 1–7 depending on the severity of symptoms. Following a recent meta-analysis, we identified and compared the following dimensions: positive, negative, disorganized, affect, and resistance [36]. UHR individuals were assessed using SIPS, which is divided into subsections similar to the PANSS: positive, negative, disorganized, and general/affective symptoms, rated from 0 to 6. All patients were also evaluated with the Global Assessment of Functioning Scale (GAF) [37,38], which on a scale of 0–100, measures how much a person’s symptoms interfere with their daily life. Due to the common lack of compliance in the acute phase of the disease, the complete examination was administered within four weeks of admission to the hospital or the outpatient clinic.

To compare the daily doses of medication taken by the patients, we used a chlorpromazine equivalent, which is a dose of antipsychotic drugs of similar strength to 100 mg of chlorpromazine [39].

### 2.3. Methodology of Laboratory Tests

About 40 mL of venous blood was collected from each participant according to the standardized methods. All samples were collected in the morning; subjects fasted prior to blood collection. 

#### 2.3.1. Flow Cytometry Analysis 

A BD Pharm Lyse lysing buffer (BD Bioscience) was used to lyse samples of peripheral blood (PB) twice at room temperature for a total of 10 min. They were then washed in phosphate-buffered saline (PBS) with 2% fetal bovine serum (FBS; Sigma), which yielded total nucleated cells. These were stained for hematopoietic lineage markers with the following fluorescein isothiocyanate-conjugated (FITC) antibodies (Abs; all from BD Bioscience): CD2 (clone RPA-2.10), CD3 (clone UCHT1), CD14 (clone M5E2), CD16 (clone 3G8), CD19 (clone HIB19), CD24 (clone ML5), CD56 (clone NCAM16.2), CD66b (clone G10F5), and CD235a (clone GA-R2). CD45-phycoerythrin (PE) Abs (clone HI30; BD Biosciences) and either CD 34-allophycocyanin (APC) Abs (clone 581; BD Bioscience) or CD133/1-APC Abs (Miltenyi Biotec) were used simultaneously to stain the cells for panleukocytic marker CD45. The FITC-conjugated isotype controls used (all from BD Biosciences) were as follows: mouse IgG1, κ (clone MOPC-21); mouse IgG2a, κ (clone G155-178); mouse IgG2b, κ (clone 27–35); PE-conjugated isotype control mouse IgG1, κ (clone MOPC-21); and APC-conjugated isotype control mouse IgG1, κ (clone MOPC-21). We also used APC-conjugated isotype control mouse IgG1 (clone IS5-21F5; Miltenyi Biotec, Bergisch Gladbach, North Rhine-Westphalia, Germany). PBS with 2% FBS on ice for 30 min was used for staining; we then washed, resuspended, and analyzed the cells with a NAVIOS Flow Cytometer (Beckman Coulter). At least 106 events were collected from each sample. We calculated absolute numbers of both VSELs and white blood cells per 1 mL PB, individually for each patient, based on the percentage content of these cells as per flow cytometry. Beckman Coulter’s Kaluza software was used in the analysis [17,40].

#### 2.3.2. Determination of S1P by Reversed-Phase High-Performance Liquid Chromatography

We centrifuged the fresh blood samples (250× *g*) for 10 min at 20 °C, and the resultant plasma was frozen at −80 °C for further study. The plasma samples were defrosted at room temperature before the addition of the internal standard (D-erythrosphingosine-1-phosphate, S1P C17, Avanti Polar Lipids), 1 M NaCl, methanol, and 37% HCl; they were then vortexed. We added 2 mL of chloroform to the samples, mixed them in a test tube rotator, and centrifuged them; the lower chloroform phase was then transferred into a new tube. Re-extraction with another 2 mL of chloroform was performed, and then a SpeedVac was used to combine and vacuum-dry the chloroform phases for 45 min at 45 °C. The residue after evaporation was reconstituted in methanol, incubated with o-phthaldialdehyde, methanol, mercaptoethanol, and boric acid (pH 10.5), then centrifuged. The supernatant was analyzed with a Hewlett Packard Series 1200 chromatograph, and the data from the chromatograph were processed using HP Chemstation software (Hewlett Packard, now Agilent). Reversed-phase high-performance liquid chromatography was performed with a Cosmosil C18-ARII column (250 mm × 4 mm, 5 mm; Phenomenex) with a C18-ARII cartridge (10 mm × 4.6 mm) packed with the same material. The column temperature was 25 °C. The mobile phase was made up of K2HPO4 (pH 5.5) and methanol (15:85; *v/v*) using an isocratic method. The flow rate was 1 mL/min, and 50 mL samples were injected every 30 min. A fluorescence detector was used for detection at 340 nm excitation and 455 nm emission wavelengths. Quantitation was based on peak areas with internal standard calibration [41,42,43,44].

#### 2.3.3. Real-Time Quantitative Reverse-Transcription Polymerase Chain Reaction

Total RNA was isolated from lysed blood with the RNeasy Kit (QIAGEN). Oligo-dT primers and the FirstStrand cDNA Synthesis Kit (Fermentas) were used to reverse transcribe the RNA. A quantitative assessment of mRNA levels was obtained with real-time RT-PCR on an ABI 7500 Fast instrument with Power SYBR Green PCR Master Mix reagent. Real-time conditions were as follows: 95 °C (15 s), 40 cycles at 95 °C (15 s), and 60 °C (1 min). Melting point analysis showed that only one PCR product was amplified under these conditions. The quantity of a target relative to a calibrator, normalized to the endogenous control β-2 microglobulin gene, is given by 2^−ΔΔCt^ (fold difference), where Ct is the threshold cycle, ΔCt = (Ct of target genes) − (Ct of endogenous control gene, β-2 microglobulin), and ΔΔCt = (ΔCt of samples for target gene) − (ΔCt of the calibrator for the target gene) [17,45].

#### 2.3.4. Determination of C5b-9, C3a, and C5a Complement Components and SDF-1

The Human C5b-9 ELISA Kit, Human C3ac ELISA Kit, and Human C5a ELISA Kit (BD OptEIA) were used to determine C5b-9 (membrane attack complex), C3a, and C5a complement components, respectively. The Human CXCL12/SDF-1 ELISA Kit (R&D Systems) was used to determine SDF-1 [40,43,46,47].

### 2.4. Statistical Analysis

The statistical analysis was performed using IBM SPSS 27 (IBM Corp., Armonk, NY, USA). Continuous variables were described in terms of means (*M*) and standard deviations (*SD*). Normality of distributions was assessed with the Shapiro–Wilk test and skewness and kurtosis values: skewness from −2 to +2 and kurtosis from −7 to +7 was taken as indicating a normal distribution [48]. Age was normally distributed in all groups. Global functioning assessed with the GAF and psychopathology dimensions assessed by the PANSS or SIPS were normally distributed for the two clinical groups. However, illness duration, chlorpromazine equivalent, and exacerbation were not normally distributed. SDF-1 and S1P were normally distributed for all three groups, whereas other biological parameters were normal only in the UHR or FEP groups. Differences between the two groups were examined with Student’s *t*-test when the appropriate conditions were met; the Mann–Whitney *U* test was used otherwise. One-way analysis of variance (ANOVA) was used to assess differences between three groups when the relevant conditions were met, and the Kruskal–Wallis *H* test was used if they were not met. Comparisons between groups were made with the Games–Howell post hoc test (for parametric tests) and Dunn’s post hoc test (for non-parametric tests). Cohen’s *d* or η^2^ (parametric tests) [49] and Wendt’s *r_U_* or *E*^2^ (non-parametric tests) [50] were used to determine the magnitudes of effect sizes for differences between groups. Differences in proportions between groups were checked using the chi-squared test for cross-tabulation with Bonferroni correction; effect size was measured with Cramér’s V correlation. Finally, Pearson’s *r* or Spearman’s *rho* correlation coefficients were estimated to assess relationships between biological parameters and psychopathological symptoms in the two clinical groups. All analyses were corrected for multiple comparisons using the Holm–Bonferroni method. The value of alpha was set to 0.05, and all analyses had a statistical power above 0.80 [49].

## 3. Results

### 3.1. Participant Characteristics

Demographic and clinical characteristics are presented in Table 1. 

There were no significant differences between the three groups in terms of age or sex. Moreover, there were no significant differences between the two clinical groups in duration of illness or exacerbation. However, there were significant differences in the values of chlorpromazine equivalent (*p* < 0.001) and global functioning measured via the GAF (*p* = 0.042). Moreover, there were group differences in types of medication: more FEP patients had atypical medications (*p* < 0.05) and more UHR individuals had no treatment (*p* < 0.05).

### 3.2. Differences in Biological Parameters

As can be seen in Table 2, there were significant differences in the C3a parameter (*p* = 0.010).

Post hoc analysis showed that UHR individuals and FEP patients had higher levels of C3a than HC participants (*p* = 0.011 and *p* = 0.040, respectively). There were no significant differences between all three groups in other biological parameters.

### 3.3. Relationships between Biological Parameters and Psychopathology Dimensions

As can be seen in Table 3, in UHR individuals there were no significant correlations between the biological parameters and psychopathological dimensions measured via SIPS.

However, in FEP patients, there were significant negative correlations of positive symptoms with C5b-9 and SDF-1 (both *p* = 0.012, but after Holm–Bonferroni *p*-value correction, these correlations were not significant). There was also a significant positive correlation between VSELs (CD133+) and negative symptoms in this group (*p* = 0.002; after Holm–Bonferroni *p*-value correction, this correlation was still significant, *p* = 0.018).

## 4. Discussion

This study compared the levels of hematopoietic stem cells (HSCs), very small embryonic-like stem cells (VSELs), and factors affecting the trafficking thereof (SDF-1, S1P, C3a, C5a, and C5b-9) among ultra-high risk (UHR) individuals, patients with first-episode psychosis (FEP), and healthy controls (HC). We hypothesized that the levels of stem cells (SCs) or the aforementioned factors would enable us to distinguish UHR and FEP groups from HC. Our results revealed increased levels of C3a in the UHR and FEP groups, compared with HC, which is in contrast with the results of previous research [26]. The scarcity of studies in this field makes interpretation of the data difficult. The complement cascade (CC) is involved in both innate and adaptive immunity and it plays an important role in stem cell mobilization [51,52] and synaptic pruning, both in normal brain development and pathological conditions, such as neuroinflammatory disorders [53]. Evidence supporting various CC dysregulations in schizophrenia has been reported [54]; however, a recent meta-analysis showed no significant differences in levels of C3 and C4 between schizophrenia patients and controls, but in most of the analyzed research, active forms of CC were not measured [55].

Complement cleavage fragments C3a and C5a are anaphylatoxins that control the local pro-inflammatory response by stimulating chemotaxis and activation of leucocytes [56], resulting in, inter alia, secretion of IL-1, IL-6, and vasoactive amines, and leading to increased vascular permeability and vasodilatation. They have also been shown to affect the blood–brain barrier (BBB), which in healthy humans prevents the access of plasma proteins to the brain [57,58]. Leakage of the BBB resulting in microglial activation and cytokine production may alter various processes, such as neurogenesis, neurotransmitter function, and white matter function, and contribute to schizophrenia symptoms, such as cognitive deterioration [59,60]. Increased C3a might, therefore, support the role of inflammation in the etiology of schizophrenia. On the other hand, there are also reports that C3a and C5a fragments play neuroprotective roles [61,62]. Data also shows that, in mice, C3a itself stimulates neurogenesis, both directly and indirectly, by inducing neural growth factor expression in microglial cells and IL-6 mRNA expression. It is also involved in the differentiation, maturation, and migration of hematopoietic and neural progenitor cells induced by SDF-1 α [63] and, therefore, plays an important role in regeneration processes. 

We did not find any significant differences between the groups regarding VSEL, HSC, S1P, or SDF-1, which might be due to the small sample size. There are reports of increased numbers of VSELs in peripheral blood and decreased plasma concentration of S1P [26] or SDF-1 [64]. However, because of the scarcity of studies, this area requires further research on larger groups of patients.

There are also scarce data on the numbers of SCs and factors affecting the mobilization thereof in other psychiatric disorders. In patients with bipolar disorder (BD) not taking lithium, there are reports of both an increased number of VSELs [27] and no differences in levels of SCs between patients with bipolar disease (BD) and healthy controls but increased numbers of VSELs in patients with BD type I [65]. Reginia et al. also showed increased numbers of complement cascade components C3a, C5a, and C5b-9 in BD patients [66]. In contrast, patients with panic disorder showed lower levels of HSCs and factors responsible for SC movement (C3a, C5a, C5b, S1P, and SDF-1) when compared to the control group, and a lower number of VSELs before treatment when compared with patients after treatment [28]. These studies suggest that SCs and factors affecting the mobilization thereof might be useful in differentiating between psychotic and stress-related disorders. They also suggest the possible impact of treatment on neurogenesis, which requires further research.

The second aim of this study was to assess whether there is any relationship between levels of SCs and factors affecting the trafficking thereof with the severity of illness symptoms. No significant correlations were found in the UHR group, which may be due to the small sample size or group selection, as we did not divide UHR individuals into subgroups based on the duration and severity of their symptomatology. In FEP patients, we found a significant positive correlation between VSELs and negative symptoms, which, to our knowledge, is a novel finding. Negative symptoms (i.e., blunted affect, emotional and social withdrawal, and poor rapport) are one of the core domains of schizophrenia and are commonly seen in patients; even up to 90% of FEP patients present at least one negative symptom, and 73% of patients have experienced them before the onset of positive symptoms [67]. Current evidence points to dopamine and glutamate as the most probable causes of negative symptoms; thus, some studies suggest that inflammatory markers such as IL-6 or C-reactive protein play a role in their etiology [68]. Additionally, specific changes in brain volume or density may potentially be involved in the manifestation of negative symptoms, as well as mobilization of SCs and regeneration processes. An increased number of VSELs has previously been linked to neuronal loss in stroke patients and a correlation has been discovered between stroke extensiveness, SDF-1 concentration in serum, and the number of VSELs in the peripheral blood [17]. Despite the growing volume of research investigating neural correlates of negative symptoms, the results have been inconsistent, and a variety of changes have been implicated, such as gray matter reduction in the cerebellum or right posterior cingulate, or white-matter reduction in areas near the anterior cingulate and right internal capsule. Furthermore, correlations have been found between negative symptoms and fractional anisotropy (the most commonly analyzed parameter, used in diffusion imaging as a measure of white matter integrity) in, inter alia, the internal capsule, anterior thalamic radiation, superior longitudinal fasciculus, fronto-occipital fasciculus, and corpus callosum [69]. Finally, it may be necessary to distinguish between primary negative symptoms (caused by the underlying pathophysiology) and secondary negative symptoms (related to, e.g., depression, antipsychotic treatment, or medical comorbidities, such as multiple sclerosis, Parkinson’s disease, or traumatic brain injury).

These results should be treated with caution for several reasons. First of all, our research is a cross-sectional study on a small number of participants. Psychiatric diagnostics and treatment are still considered stigmatizing. Therefore, even though the duration of untreated psychosis is one of the most significant prognostic markers, predicting, inter alia, poorer global functioning [70], its value is underestimated, as people tend to only seek treatment when they are experiencing intense personal distress. Additionally, the low reporting rate might be affected by the type of psychopathology (i.e., poor insight, or initially non-specific and intermittent symptoms). Moreover, the UHR group is a highly heterogeneous group, including individuals with subthreshold attenuated positive symptoms, transient psychotic symptoms, genetic risk, and deterioration, each characterized by different duration and severity of symptoms, as well as transition risk [71]. In our study, only two UHR individuals developed full-blown psychosis, which is not enough to draw any conclusions. Hence, longitudinal studies on larger groups of at-risk patients, possibly including first-episode psychosis and chronic schizophrenia, are required. Another limitation is that not all our patients were drug-naïve. Thus, we cannot exclude the potential influence of drugs on levels of SCs and factors affecting their trafficking, and there are reports of the influence of antidepressant and antipsychotic drugs on neurogenesis [72,73,74]. Additionally, potential confounders such as diet, physical activity, and smoking should be taken into consideration. 

## 5. Conclusions

In conclusion, our research showed increased levels of C3a in the UHR and FEP groups when compared with HC. We did not find any significant differences in the levels of HSC, VSEL, C5a, S1P, or SDF-1 in either the UHR or FEP groups. However, a significant positive correlation between VSELs (CD133+) and negative symptoms was found among FEP patients. These results, although preliminary, support the role of the immune system and the disturbance of regenerative processes in the etiology of schizophrenia. The study of stem cells and factors associated with their movement seems promising in diagnosing schizophrenia and differentiating it from other psychiatric disorders. Due to the small sample size, further research on individuals from each stage of the schizophrenia spectrum is necessary to establish the relevance of C3a and other factors as potential biomarkers of this disabling disease.

## Figures and Tables

**Table 1:** Demographic and clinical characteristics of participants from the three groups.

Variable	Ultra-High Risk Individuals (UHR; *n* = 9)	First-Episode Psychosis Patients (FEP; *n* = 22)	Healthy Controls (HC; *n* = 18)	*F*/*t*/*Z*/χ^2^	*r_U_*/*V*
Age: *M* (*SD*)	25.00 (5.52)	28.27 (6.12)	30.33 (6.13)	2.37 ^a^	-
Sex: female/male	6/3	11/11	10/8	0.72 ^b^	-
Antipsychotic medications:					
Atypical: *n* (%)	3 (33.33)	19 (86.36)	-	18.43 ^b^ ***	0.77 ^e^
Atypical and typical: *n* (%)	0 (0.00)	2 (9.09)	-		
Typical: *n* (%)	0 (0.00)	1 (4.55)	-		
No medications: *n* (%)	6 (66.67)	0 (0.00)	-		
Chlorpromazine equivalent (mg): *M* (*SD*)	50.44 (101.65)	479.09 (247.42)	-	−4.05 ^c^ ***	0.93 ^f^
Duration of illness: *M* (*SD*)	0.98 (1.54)	0.84 (1.47)	-	−0.44 ^c^	-
Exacerbation: *M* (*SD*)	3.56 (5.22)	1.05 (0.21)	-	−1.57 ^c^	-
Global functioning (GAF): *M* (*SD*)	70.56 (8.88)	54.73 (17.18)	-	−2.51 ^d^ *	0.58 ^g^
Psychopathology dimensions (SIPS):					
Positive symptoms: *M* (*SD*)	5.00 (3.74)	-	Min–Max = 2.00–13.00		
Negative symptoms: *M* (*SD*)	8.25 (5.70)	-	Min–Max = 1.00–15.00		
Disorganization: *M* (*SD*)	3.50 (3.21)	-	Min–Max = 1.00–9.00		
General symptoms: *M* (*SD*)	6.25 (4.20)	-	Min–Max = 0.00–14.00		
Psychopathology dimensions (PANSS):					
Positive Symptoms: *M* (*SD*)	-	12.27 (4.33)	Min–Max = 5.00–22.00		
Negative Symptoms: *M* (*SD*)	-	18.32 (7.07)	Min–Max = 7.00–35.00		
Disorganization: *M* (*SD*)	-	15.09 (4.50)	Min–Max = 8.00–23.00		
Affect: *M* (*SD*)	-	10.18 (4.19)	Min–Max = 5.00–21.00		
Resistance: *M* (*SD*)	-	5.32 (1.94)	Min–Max = 4.00–11.00		

PANSS = Positive and Negative Syndrome Scale. SIPS = Structured Interview for Psychosis-Risk Syndromes. GAF = Global Assessment of Functioning. ^a^ One-way analysis of variance *F* test. ^b^ Chi-squared test. ^c^ Mann–Whitney *U*. ^d^ Student’s *t*-test. ^e^ Cramér’s V correlation effect size. ^f^ Wendt’s *r* rank-biserial correlation effect size. ^g^ Cohen’s *d* effect size. * *p* < 0.05. *** *p* < 0.001.

**Table 2:** Comparison of biological parameters between participants from the three groups.

Variable	Ultra-High Risk Individuals (UHR; *n* = 9)	First-Episode Psychosis Patients (FEP; *n* = 22)	Healthy Controls (HC; *n* = 18)	*H*/*F*	*E* ^2^
VSELs (CD34^+^): *M* (*SD*)	0.09 (0.09)	0.12 (0.10)	0.08 (0.07)	3.19 ^a^	-
HSC (CD34^+^): *M* (*SD*)	0.79 (0.31)	1.30 (1.31)	1.34 (0.80)	3.88 ^a^	-
VSELs (CD133^+^): *M* (*SD*)	0.03 (0.01)	0.04 (0.07)	0.04 (0.03)	0.11 ^a^	-
HSC (CD133^+^): *M* (*SD*)	0.79 (0.98)	0.76 (0.60)	0.54 (0.43)	2.04 ^a^	-
C3a: *M* (*SD*)	400.65 (191.43) ^d^ *	454.52 (548.84) ^e^ **	191.42 (74.71)	13.33 ^a^ **	0.27 ^c^
C5a: *M* (*SD*)	6.09 (5.25)	6.07 (6.86)	3.88 (1.56)	3.06 ^a^	-
C5b-9: *M* (*SD*)	350.20 (178.34)	361.23 (152.27)	332.98 (151.51)	0.91 ^a^	-
SDF-1: *M* (*SD*)	2819.11 (976.18)	2914.05 (523.69)	3237.94 (964.65)	1.15 ^b^	-
S1P: *M* (*SD*)	8.48 (1.09)	7.82 (0.82)	8.05 (0.68)	2.08 ^b^	-

VSELs = very small embryonic-like stem cells. HSCs = hematopoietic stem cells. C3a, C5a, C5b-9 = complement cascade cleavage fragments 3a, 5a, 5b-9. SDF-1 = stromal-derived factor 1. S1P = sphingosine-1-phosphate. ^a^ Kruskal–Wallis *H* test. ^b^ One-way analysis of variance *F* test. ^c^ Epsilon squared effect size. All *p*-values for post hoc: ^d^ UHR patients vs. HC participants. ^e^ FEP patients vs. HC participants. * *p* < 0.05. ** *p* < 0.01.

**Table 3:** Relationship between biological parameters and psychopathological dimensions in the two clinical groups.

**Variable**	**Psychopathological Dimensions**							
	**SIPS—Positive Symptoms**		**SIPS—Negative Symptoms**		**SIPS—Disorganization**		**SIPS—General Symptoms**	
**Ultra-High Risk Individuals (UHR; *n* = 9)**								
	*r*/*rho*		*r*/*rho*		*r*/*rho*		*r*/*rho*	
VSELs (CD34^+^)	0.70 ^a^		0.08 ^a^		−0.09 ^a^		0.45 ^a^	
HSC (CD34^+^)	0.31 ^a^		−0.07 ^a^		−0.36 ^a^		0.18 ^a^	
VSELs (CD133^+^)	0.31 ^a^		−0.47 ^a^		−0.37 ^a^		−0.25 ^a^	
HSC (CD133^+^)	−0.43 ^b^		−0.12 ^b^		−0.18 ^b^		0.23 ^b^	
C3a	0.28 ^a^		−0.36 ^a^		−0.17 ^a^		−0.23 ^a^	
C5a	−0.07 ^b^		0.10 ^b^		0.10 ^b^		−0.18 ^b^	
C5b-9	0.24 ^a^		−0.47 ^a^		−0.32 ^a^		−0.54 ^a^	
SDF-1	−0.05 ^a^		0.04 ^a^		0.36 ^a^		0.06 ^a^	
S1P	−0.32 ^a^		0.10 ^a^		0.06 ^a^		0.17 ^a^	
	**Psychopathological dimensions**							
**Variable**	**PANSS—Positive Symptoms**	**PANSS—Negative Symptoms**		**PANSS—Disorganization**		**PANSS—Affect**		**PANSS—Resistance**
**First-episode Psychosis Patients (FEP; *n* = 22)**								
	*r*/*rho*	*r*/*rho*		*r*/*rho*		*r*/*rho*		*r*/*rho*
VSELs (CD34^+^)	0.17 ^b^	0.19 ^b^		0.27 ^b^		0.24 ^b^		−0.15 ^b^
HSC (CD34^+^)	0.13 ^b^	−0.17 ^b^		−0.21 ^b^		0.24 ^b^		0.23 ^b^
VSELs (CD133^+^)	−0.01 ^b^	0.63 ^b^ **^c/^*^d^		0.42 ^b^		−0.04 ^b^		−0.22 ^b^
HSC (CD133^+^)	0.31 ^b^	−0.06 ^b^		−0.16 ^b^		0.29 ^b^		0.21 ^b^
C3a	0.16 ^b^	0.17 ^b^		0.20 ^b^		0.00 ^b^		0.22 ^b^
C5a	0.09 ^b^	−0.16 ^b^		−0.28 ^b^		−0.19 ^b^		−0.06 ^b^
C5b-9	−0.53 ^b^ *^c^	−0.14 ^b^		−0.26 ^b^		−0.22 ^b^		−0.27 ^b^
SDF-1	−0.52 ^a^ *^c^	0.04 ^a^		−0.25 ^a^		−0.25 ^a^		−0.13 ^b^
S1P	0.12 ^a^	−0.28 ^a^		−0.04 ^a^		0.07 ^a^		0.03 ^b^

PANSS = Positive and Negative Syndrome Scale. SIPS = Structured Interview for Psychosis-Risk Syndromes. VSELs = very small embryonic-like stem cells. HSCs = hematopoietic stem cells. C3a, C5a, C5b-9 = complement cascade cleavage fragments 3a, 5a, 5b-9. SDF-1 = stromal-derived factor 1. S1P = sphingosine-1-phosphate. ^a^ Pearson’s *r* correlation coefficient. ^b^ Spearman’s *rho* correlation coefficient. ^c^ *p*-value before Holm–Bonferroni correction. ^d^ *p*-value after Holm–Bonferroni correction. * *p* < 0.05. ** *p* < 0.01.

## Data Availability

Materials from the study reported here are available from the corresponding author upon reasonable request.
